# Determinants of pain intensity and magnitude of incapability more than two years after arthroscopic Bankart repair for anterior shoulder instability

**DOI:** 10.1016/j.jseint.2024.05.001

**Published:** 2024-05-17

**Authors:** Melle M. Broekman, Lukas P.E. Verweij, Job N. Doornberg, Sebastiaan Floor, David Ring, Michel P.J. van den Bekerom

**Affiliations:** a Department of Surgery and Perioperative Care, Dell Medical School at the University of Texas at Austin, Austin, TX, USA; b Faculty of Behavioural and Movement Sciences, Vrije Universiteit van Amsterdam, Amsterdam, The Netherlands; c Department of Orthopaedic Surgery, Shoulder and Elbow Unit, Amsterdam, The Netherlands; d Department of Orthopedic Trauma Surgery, Universitair Medisch Centrum Groningen, Groningen, The Netherlands; e Department of Orthopedic Surgery, Centraal Militair Hospitaal, Utrecht, The Netherlands

**Keywords:** Bankart repair, Mental health, Pathophysiology severity, Unhelpful thinking, Incapability, Pain intensity

## Abstract

**Background:**

Individuals treated with arthroscopic Bankart repair after anterior shoulder dislocations experience varied discomfort and incapability. The aim of this study was to determine the relative association of mental health and physical health factors with 1) magnitude of capability and 2) pain intensity 2 or more years after surgery.

**Methods:**

This cross-sectional study evaluated 80 military patients that experienced one or more traumatic anterior shoulder dislocations a minimum of 2 years after arthroscopic Bankart repair without remplissage. We measured capability (Oxford Shoulder Instability Score), pain intensity using an 11-point ordinal scale, symptoms of anxiety (Generalized Anxiety Disorder-2 questionnaire), symptoms of depression (Patient Health Questionnaire-2), catastrophic thinking (Pain Catastrophizing Scale-4), and kinesiophobia (Tampa scale for kinesiophobia-4). We also identified preoperative presence of a Hill-Sachs lesion on radiographs and postoperative occurrence of subluxation or a dislocation episode. A negative binominal regression analysis sought factors associated with magnitude of incapability and pain intensity.

**Results:**

Greater incapability was strongly associated with both greater kinesiophobia (Regression Coefficient [RC] = −0.50; 95% confidence interval [CI] = −0.73 to −0.26; *P* ≤ .01) and repeat surgery (RC = −0.27; 95% CI = −0.41 to −0.13; P ≤ .01). Greater pain intensity was only strongly associated with greater kinesiophobia (RC = 0.25; 95% CI = 0.039 to 0.46; *P* = .021).

**Conclusion:**

The observation that greater unhelpful thinking is associated with greater pain intensity and greater magnitude of incapability after a Bankart repair for anterior shoulder instability, whereas pathophysiological factors such as glenoid bone loss were not, emphasizes the degree to which mindset is associated with musculoskeletal health.

## Background

Active young people can be offered surgical treatment after their first anterior shoulder dislocation due to a high risk of recurrent dislocation.^16^^,^^20^ Among people who decline immediate operative treatment, later reasons to consider offering surgical treatment include more than one radiographically documented anterior shoulder dislocation or, more debatably, symptoms suggestive of episodic subluxation in the absence of documented dislocation.^16^^,^^24^ The results of surgery can be measured objectively, such as number of dislocation episodes, and subjectively, such as perceived subluxation episodes, pain intensity, and capability, including confidence to return to activity which are measured using Patient Reported Outcome Measures (PROMs). There is mounting evidence that variation in pain intensity and magnitude of capability (PROM scores) is less attributed to pathophysiology severity, relative to psychosocial factors.^7^^,^^28^

### Rationale

For instance, a study of patients seeking care for rotator cuff pathophysiology found that magnitude of incapability is associated with greater symptoms of depression, but not with measures of pathophysiology severity such as defect size or degree of retraction.^18^ Another review of factors associated with magnitude of incapability after upper extremity injuries found that depression, catastrophic thinking, and pain self-efficacy were most consistently associated with incapability, more so than measures of impairment such as injury severity.^11^ In the case of shoulder dislocations, one study suggests that patients with a good anatomical result and no repeat dislocations after surgical treatment for shoulder instability often have more discomfort and less capability than expected.^2^ Separate studies of reconstruction of the anterior cruciate ligament in the knee found similar results, but also showed that mental health factors such as catastrophic thinking and fear of painful movement might, in part, explain this greater incapability.^6^^,^^10^^,^^27^ Perhaps young athletes and soldiers feel incapable in spite of a stable shoulder due to unhelpful thoughts (e.g. misinterpretation of symptoms as represented by measures such as kinesiophobia) and emotions (worry and despair) regarding the initial injury and the changes to the shoulder, as well as dread of the possibility of future subluxation or dislocation and possible consequences.^12^^,^^17^^,^^23^ In other words, it’s possible that mental health factors might have a stronger association with discomfort and incapability after surgery for anterior shoulder instability, more so than objective measures such as laxity, stiffness, arthritis, and glenoid bone loss.

Therefore, we sought to determine which pathophysiological and psychosocial factors are associated with: 1) variation in magnitude of capability measured by the Oxford Shoulder Instability Score (OSIS) and 2) variation in pain intensity measured by the Brief Pain following an arthroscopic Bankart repair with minimum 2-year follow-up.

## Methods

### Study design and setting

An IRB-approved, cross-sectional study included military patients with a first-time traumatic anterior shoulder dislocation who were treated with an arthroscopic Bankart repair without remplissage by one of three surgeons at a Dutch military urban hospital between 2011 and 2019. This manuscript was written according to the Strengthening the Reporting of Observational Studies in Epidemiology checklist for cross-sectional studies. Informed consent was obtained by each patient in this study.

### Participants

Military staff aged 18 years of age or older with a first-time traumatic anterior shoulder dislocation treated with arthroscopic Bankart repair without remplissage between 2011 and 2019 and a minimum follow-up of 2 years after surgery were invited to participate. Patients were excluded if they were treated for concomitant injures which required surgery, a rotator cuff tear, nerve injuries, Humeral Avulsion Glenohumeral Ligament repair, shoulder arthroplasty, or a posterior labrum repair. All eligible patients were asked to complete an online questionnaire using an electronic data management system (Castor EDC, New York, NY, USA). Among the 122 eligible patients, 112 (92%) responded to the email or phone call and agreed to participate, 7 (6%) could not be contacted and 3 (2%) declined participation. Patients that refused believed the study did not have additional benefit for them, and therefore did not want to participate. After sending final reminders through email, 80 patients completed the online survey which resulted in an overall response rate of 66%. The mean age was 27 years ± 6 years (Table I). Sixty-six percent of the patients (N = 53) were in active military service, 85% (N = 68) had a Hill Sachs lesion, and 21 percent (N = 17) experienced a postoperative dislocation. All patients had on-track lesions.

**Table I:** Demographics of cohort.

Variables	Value∗
N	80
Age	27 ± 5.9
Gender	
Male	73 (91%)
Female	7 (9%)
% Glenoid bone loss	27% (0 to 94)
Follow-up in weeks	232 (173 to 360)
Active military	
Yes	53 (66%)
No	27 (34%)
Revision surgery	
Yes	7 (9%)
No	73 (91%)
Hill Sachs lesion	
Yes	68 (85%)
No	12 (15%)
Postoperative dislocation	
Yes	17 (21%)
No	63 (79%)
Number of anchors used	
1	5
2	72
3	3
Sports level	
Amateur	41
Competitive	35
Professional	4

∗ Value is displayed as median with interquartile range for continuous nonparametric variables, as mean with standard deviation for continuous variables with normal distribution, and as number with percentage for categorial variables.

### Primary outcome measures

Magnitude of incapability was measured using the OSIS.^5^ The OSIS is a short, 12-item, condition-specific, PROM developed to measure capability among people with unidirectional or multidirectional instability of the shoulder. Scores range from 0 to 48 with lower scores representing greater magnitude of incapability.

Pain intensity was measured using a Numeric Rating Scale (NRS) for Pain from the Brief Pain Inventory, and 11-point ordinal scale from 0 representing no pain to 10, the worst pain imaginable.^22^

### Explanatory variables

We measured symptoms of anxiety using the Generalized Anxiety Disorder-2 questionnaire (GAD-2) and symptoms of depression using the Patient Health Questionnaire-2.^1^ Unhelpful thinking was measured using the 4-question versions of both the Pain Catastrophizing Scale (PCS) and the Tampa scale for kinesiophobia.^14^

Other explanatory variables included patient report of a postoperative subluxation event (yes/no), repeat surgery for one or more repeat dislocation after primary surgery (yes/no), percentage of preoperative glenoid bone loss calculated from computed tomography and magnetic resonance imaging scans on a continuum by one of the participating surgeons, preoperative presence of a Hill-Sachs lesion on radiographs or magnetic resonance imaging scans judged by one of the participating surgeons (yes/no), age at the time of operation, follow-up length in weeks, gender, level of sports (amateur, competitive, professional), number of anchors used, and active military service (yes/no).

### Statistical analysis

We performed descriptive statistics where continuous data with normal distribution was described as mean with standard deviation, continuous nonparametric data was described as median with interquartile range, and categorical data was described in numbers with percentage. Mann-Whitney U tests and Kruskal-Wallis H tests were used for nonparametric continuous data (Appendix 1 and 2). The Spearman correlation coefficient was calculated to assess the correlation between all continuous explanatory variables and 1) OSIS score, and 2) NRS pain intensity score (Appendix 1 and 2). Spearman correlations suggested that GAD-2, PCS, and the Tampa scale for kinesiophobia were multicollinear.^21^ After running separate regression analyses of factors associated with both capability and pain intensity for all three variables, the Tampa scale for kinesiophobia was found to have the best model fit based on the Akaike Information Criterion Score, and GAD-2 and PCS were excluded from analysis. All variables with a *P* value below .10 in bivariate analysis were moved to multivariable analysis, excluding all variables with a *P* value above .10 to account for potential confounders. A negative binominal regression analysis was used to seek factors associated with the OSIS score and NRS pain intensity score. Associations were reported as a Regression Coefficient (RC) with a 95% confidence interval and Standard Error. All variables with a *P* value below .05 were considered statistically significant. There was no missing data.

## Results

### Factors associated with variation in magnitude of incapability

Accounting for potential confounding among variables that were significant in bivariate analysis such as symptoms of anxiety and symptoms of depression, greater incapability measured by the OSIS was strongly associated with greater unhelpful thinking measured by the Tampa scale for kinesiophobia (RC = −0.50; 95% confidence interval [CI] = −0.73 to −0.26; *P* ≤ .01), and strongly associated with revision surgery (RC = −0.27; 95% CI = −0.41 to −0.13; *P* ≤ .01; Table II).

**Table II:** Negative binominal regression analysis of factors associated with magnitude of incapability.

	Regression coefficient (95% confidence interval)∗	Standard error	*P* value
Mental factors			
PHQ score	−0.0054 (−0.049 to 0.038)	0.022	.81
Tampa scale	−0.50 (−0.73 to −0.26)	0.11	**<.01**
Pathophysiological factors			
Revision surgery	−0.27 (−0.41 to −0.13)	0.070	**<.01**

*PHQ*, patient health questionnaire.

Bold indicates statistical significance, *P* < .05. Factors with *P* < .10 in bivariate analysis were moved into negative binominal regression analysis.

∗ Effect sizes can be interpreted as strong for coefficients < −0.15.

### Factors associated with variation in pain intensity

Accounting for potential confounders such as a Hill-Sachs lesion, symptoms of anxiety and symptoms of depression, greater pain intensity measured by the NRS for pain was strongly associated with greater unhelpful thinking measured by the Tampa scale for kinesiophobia (RC = 0.25; 95% CI = 0.039 to 0.46; *P* = .020; Table III).

**Table III:** Negative binominal regression analysis of factors associated with variation in pain intensity.

	Regression coefficient (95% confidence interval)∗	Standard error	*P* value
Mental factors			
PHQ score	−0.94 (−0.62 to 0.43)	0.27	.72
Tampa scale	0.25 (0.039 to 0.46)	0.11	**.020**
Pathophysiological factors			
Hill Sachs lesion	−0.25 (−1.3 to 0.85)	0.56	.66

*PHQ*, patient health questionnaire.

Bold indicates statistical significance, *P* < .05. Factors with *P* < .10 in bivariate analysis were moved into negative binominal regression analysis.

∗ Effect sizes can be interpreted as strong for coefficients >0.10.

## Discussion

Orthopedic surgeons may expect symptom severity to correspond with the severity of the disease (pathophysiology). In other words, the level of pain, and the difficulty with daily tasks (incapability) may be expected to correlate with the severity of radiographic arthritis, limitations of motion, and other objective measures of pathophysiology. There is mounting evidence that there is limited correspondence between pathophysiology severity and levels of discomfort and incapability in people with musculoskeletal disorders.^4^^,^^13^^,^^18^ It is somewhat counter-intuitive that the disjunction between the severity of the disease and the severity of the illness (state of being unwell) is accounted for by thoughts and emotions regarding the body’s sensations.^4^^,^^13^^,^^18^ In other words, patient reported outcomes (PROs) (levels of discomfort and incapability) are associated with mindsets in addition to measures of pathophysiology severity. Also, the association of PROs is sometimes completely unrelated to measures of pathophysiology severity such as the size of a rotator cuff defect and associated muscle atrophy and fat replacement of the rotator cuff muscles, which raises the question whether PRO’s are primarily measuring the quality of surgical restoration of anatomy, or whether they are measuring one’s thoughts and emotions regarding their musculoskeletal disorder. Among young athletes, recovery and return to physical roles also might be more related to mental health than severity of pathophysiology. In a young military cohort treated with an arthroscopic Bankart repair for anterior shoulder dislocation we sought factors associated with variation in magnitude of capability and pain intensity more than 2 years after surgery. Our most important finding was that greater pain intensity and incapability are largely associated with greater fear of movement, and not with pathophysiological factors such as glenoid bone loss or more than one postoperative dislocation.

This study had several limitations. First, the cross-sectional design of the study cannot test direction of association or causation between mental health, capability and pain intensity since all data was collected at one point in time without follow-up. In our opinion, knowledge of associations in cross-sectional cohorts is sufficient to help guide health strategies and direction of those associations is less relevant. Ideally, mental health status would also be measured preoperatively to further understand the association of preoperative mental health status with postrecovery comfort and capability. For instance, prior studies of arthroplasty patients found that worse preoperative mental health (symptoms of depression and lower short form health survey-36 Mental Component Scores) associates with greater incapability and pain intensity.^3^^,^^25^^,^^26^ Second, the call-back design of the study made it vulnerable to participation bias since a subset of patients from the registry agreed to participate. On the other hand, we studied relationships rather than rates and there seems to be sufficient variability in the variables to identify relationships that are likely reproducible in other samples. Third, the limited number of surgeons and the context may limit generalizability. On the other hand, homogeneity in both patients and surgeon factors might be a benefit by virtue of limited variation in technical factors. Also small alterations in technical factors are unlikely to influence the results, and mental health factors are common to all humans.

The observation that greater magnitude of incapability was associated with greater unhelpful thinking and revision surgery, but not with other pathophysiological factors such as the presence of a Hill-Sachs lesion or glenoid bone loss, adds to growing evidence that mental health factors predominate in recovery. Our findings align with a longitudinal cohort study among patients with traumatic and atraumatic shoulder instability that found preoperative mental health factors such as the RAND 36 Mental Component Score and comorbid formal diagnosis of major depression are associated with worse preoperative American Shoulder and Elbow Surgeon scores.^15^ Another study of patients undergoing an arthroscopic rotator cuff repair also found preoperative mental health status measured by the veterans RAND-12 Mental Component Score to be associated with postoperative American Shoulder and Elbow Surgeons scores, suggesting that baseline mental health status has an impact on postoperative recovery.^9^ Our results are also supported by a cross-sectional study of people recovering from an isolated proximal humerus fracture, which found that kinesiophobia measured within a week of injury correlates with greater magnitude of incapability six to nine months after injury.^12^ The association between lower capability and revision surgeries may reflect greater pathophysiology severity, but revision surgery might also be associated with worse mental health status.

The observation that greater pain intensity was associated with greater unhelpful thinking is in line with evidence showing the impact of mindset on recovery trajectory after surgery of the shoulder.^3^^,^^12^^,^^19^ For instance, one study of patients who underwent shoulder arthroplasty found that patients with worse preoperative mental health scores were less likely to gain a minimum clinically important difference for pain intensity and incapability.^29^ Finally, a study of people recovering from fractures of the distal radius found that mental health factors early after injury are associated with greater pain intensity up to 9 months after surgery.^8^

## Conclusion

The observation that greater unhelpful thinking is associated with both greater pain intensity and greater incapability after Bankart repair for anterior shoulder dislocation in young military personnel, whereas pathophysiological factors such as glenoid bone loss were not, emphasizes the degree to which mindset is associated with musculoskeletal health. These findings support the integration of these factors into comprehensive, patient-centered care strategies for musculoskeletal patients. In future research, randomized controlled trials might evaluate the possible benefit of including these mental health factors in treatment strategies.

## Disclaimers:

Funding: No funding was disclosed by the authors.

Conflicts of interest: David Ring certifies receipt of personal payments or benefits, during the study period, in an amount of less than USD 10,000 from Skeletal Dynamics. All the other authors, their immediate families, and any research foundations with which they are affiliated have not received any financial payments or other benefits from any commercial entity related to the subject of this article.
