# Supplementary material for: Determinants of pain intensity and magnitude of incapability more than two years after arthroscopic Bankart repair for anterior shoulder instability
Source: JSES Int. 2024 May 17;8(5):941–5. doi: 10.1016/j.jseint.2024.05.001 (PMC11401566; doi:10.1016/j.jseint.2024.05.001)
Supplement: Supplementary Table S1 [file mmc1.docx]

| Appendix 1. Bivariate analysis of factors associated with OSIS. | | |
| --- | --- | --- |
| **Variables** | **Mean ± SD** | ***P* value** |
| Active military service |  | 0.81 |
| Yes | 42 ± 8.8 |  |
| No | 41 ± 7.4 |  |
| Revision |  | **0.0018** |
| Yes | 42 ± 7.0 |  |
| No | 31 ± 9.2 |  |
| Hill Sachs lesion |  | 0.12 |
| Yes | 37 ± 9.0 |  |
| No | 41 ± 7.6 |  |
| Postoperative dislocation |  | 0.46 |
| .1 | 37 ± 9.1 |  |
| .2 | 41 ± 1.0 |  |
| >3 | 33 ± 8.3 |  |
| Anchors |  | 0.16 |
| .1 | 34 ± 11 |  |
| .2 | 41 ± 7.5 |  |
| .3 | 46 ± 4.0 |  |
| Sportslevel |  | 0.65 |
| Amateur | 42 ± 7.7 |  |
| Semi-professional | 41 ± 8.3 |  |
| Professional | 43 ± 6.6 |  |
|  | **Correlation coefficient (ρ)** | **P value** |
| Age | 0.0643 | 0.29 |
| Glenoid bone loss | -0.33 | 0.51 |
| GAD | -0.29 | **<0.01** |
| PHQ | -0.32 | **<0.01** |
| PCS | -0.52 | **<0.01** |
| TSK | -0.032 | **<0.01** |
| Follow-up in weeks | -0.41 | **0.78** |
|  |  |  |
| Continuous variables as median (interquartile range). Spearman correlation indicated by ρ. GAD = Generalized Anxiety Disorder item. PHQ = Patient Health Questionnaire; PCS = Pain Catastrophizing Scale; TSK = Tampa Scale for Kinesiophobia. All variables with P<0.10 were moved to multivariable analysis. | | |
