# Supplementary material for: Determinants of pain intensity and magnitude of incapability more than two years after arthroscopic Bankart repair for anterior shoulder instability
Source: JSES Int. 2024 May 17;8(5):941–5. doi: 10.1016/j.jseint.2024.05.001 (PMC11401566; doi:10.1016/j.jseint.2024.05.001)
Supplement: Supplementary Table S2 [file mmc2.docx]

| Appendix 2. Bivariate analysis of factors associated with the Numeric Rating Scale for Pain Intensity. | | |
| --- | --- | --- |
| **Variables** | **Mean ± SD** | ***P* value** |
| Active military service |  | 0.98 |
| Yes | 0.81 ± 1.8 |  |
| No | 1.0 ± 1.5 |  |
| Revision |  | 0.49 |
| Yes | 1.1 ± 1.5 |  |
| No | 0.86 ± 1.7 |  |
| Hill Sachs lesion |  | **<0.01** |
| Yes | 1.8 ± 1.9 |  |
| No | 0.74 ± 1.6 |  |
| Postoperative dislocation |  | 0.29 |
| .1 | 1.6 ± 1.4 |  |
| .2 | 0.91 ± 0.42 |  |
| >3 | 0.60 ± 0.89 |  |
| Anchors |  | 0.55 |
| .1 | 2.4 ± 2.1 |  |
| .2 | 0.79 ± 1.6 |  |
| .3 | 0.67 ± 1.1 |  |
| Sportslevel |  | 0.43 |
| Amateur | 0.78 ± 1.8 |  |
| Semi-professional | 0.97 ± 1.6 |  |
| Professional | 1.3 ± 1.9 |  |
|  | **Correlation coefficient (ρ)** | **P value** |
| Age | -0.16 | 0.61 |
| Glenoid bone loss | 0.34 | 0.15 |
| GAD | 0.23 | **<0.01** |
| PHQ | 0.31 | **0.032** |
| PCS | 0.42 | **<0.01** |
| TSK | 0.063 | **<0.01** |
| Follow-up in weeks | 0.32 | 0.56 |
|  |  |  |
| Continuous variables as median (interquartile range). Spearman correlation indicated by ρ. GAD = Generalized Anxiety Disorder item. PHQ = Patient Health Questionnaire; PCS = Pain Catastrophizing Scale; TSK = Tampa Scale for Kinesiophobia. All variables with P<0.10 were moved to multivariable analysis. | | |
